# A Biosensor Platform for Detecting the Dissipation of Transmembrane Gradients in Single Liposomes

**DOI:** 10.1021/acs.analchem.5c02841

**Published:** 2025-09-19

**Authors:** Changcheng Zhang, Mark E. Bowen

**Affiliations:** † Department of Physics & Astronomy, 12301 Stony Brook University, Stony Brook, New York 11794-3800, United States; ‡ Department of Physiology & Biophysics, Stony Brook University, Stony Brook, New York 11794-8661, United States

## Abstract

Pore forming proteins are a diverse collection of polypeptides, which share little structural or amino acid sequence homology and span several mechanistic classes. Their commonality lies in their ability to create transmembrane pores in biological membranes, which places some pore forming proteins among nature’s most toxic substances. Such membrane pores can dissipate chemical and electrical gradients, release cellular contents, and even deliver toxic cargo. Detecting pore forming activity commonly relies on dye release assays, which measure a change in brightness as quenched dyes are diluted. Single molecule detection provides the ultimate sensitivity, but measuring relative brightness is challenging due to intensity variation across the population. An ideal sensor could allow interrogation of the entire population of liposomes after pore formation without requiring foreknowledge of the initial intensity. To achieve this we have developed a FRET biosensor approach using ligand-responsive oligonucleotides, which are encapsulated within liposomes that sustain chemical gradients. We show that dissipation of transmembrane gradients can be measured with single liposome resolution using TIRF microscopy, which allows detection of pore forming proteins regardless of mechanistic class. Our encapsulated oligonucleotide biosensors could detect the presence of Botulinum neurotoxin down to picomolar concentrations without the need for protein-specific immunoreagents and highlighted the role of proteolytic activation in pore formation by the toxin. Adapting this approach to additional oligonucleotide sensors would provide a general platform to detect transmembrane solute movement and dissect the underlying transport mechanisms.

## Graphical abstract

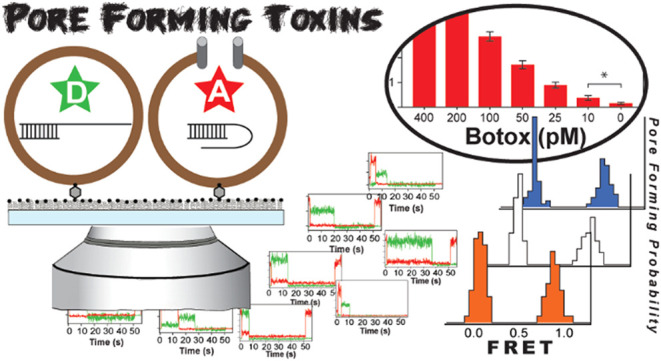

Biological phospholipid membranes form a semipermeable barrier between a cell and the environment, which supports the transmembrane gradients essential for life such as the asymmetric distribution of potassium.^1,2^ Within the eukaryotic cell, membrane-bound organelles support unique chemical environments such as the acidification within the endocytic pathway.^3^ Pore-forming proteins (PFPs) are a broad class of polypeptides that form transmembrane channels in cellular membranes allowing such gradients to dissipate.^4^ As such, pore-forming proteins play important biological roles in signal transduction, host defense, content delivery and include some of the most potent toxins known to man.^5^

Pore-forming proteins are a structurally diverse group that utilize many different mechanisms to breach the membrane.^6^ They are produced by bacteria, fungi, plants, and animals. In general, PFPs exist in a soluble form that is targeted to a specific membrane via protein or lipid receptors and then undergoes conformational changes to produce a membrane spanning pore. The simplest PFPs are membrane-active peptides, like the well-studied GALA peptide^7^ that binds to membranes and then forms oligomeric, α-helical pores at low pH (Figure 1A).^8^ Other PFPs leave residual pores in the membrane after transmembrane delivery, such as the Botulinum neurotoxin.^9^ During Botulinum intoxication, the holotoxin is proteolytically cleaved into two polypeptides that remain connected by a disulfide bridge (Figure 1B).^10^ Upon acidification, the soluble toxin undergoes a conformational change to a transmembrane state.^11^ The light chain is translocated through the membrane by the heavy chain,^12^ which leaves a residual pore in the membrane.^13,14^ The differences between GALA and Botulinum neurotoxin highlight that the unifying feature of PFPs is not structural nor mechanistic but functional in that they lead to dissipation of the same transmembrane gradients.

**Figure 1:**
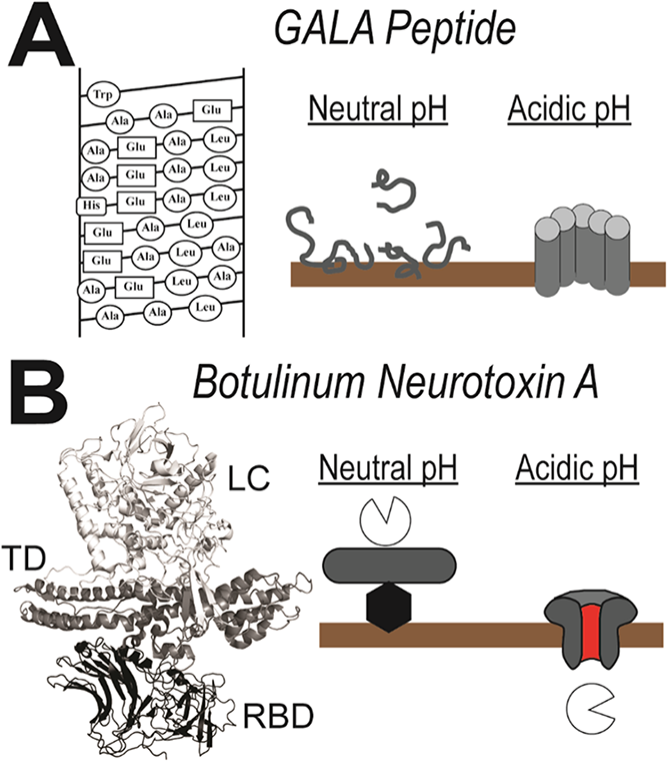
Structural and mechanistic diversity of the pore forming proteins. (A) The membrane-active GALA peptide;^7^ *Left*: Helical net representation of the amino acid sequence, showing the Glu-Ala-Leu-Ala repeats, which can adopt an amphipathic α-helical structure. *Right*: Mechanism of pore formation. At neutral pH, disordered GALA peptides (gray lines) bind the membrane (brown). At acidic pH, GALA inserts into the membrane as an oligomeric transmembrane pore. (B) Botulinum Neurotoxin A (BoNT/A); *Left*: Cartoon representation of the BoNT/A holotoxin structure (PDB ID: 3 V0C^15^), highlighting its three functional domains: receptor-binding domain (RBD, *black*), translocation domain (TD, *gray*), and enzymatic light chain (LC, *white*)^11^ *Right*: Mechanism of pore formation; At neutral pH, the BoNT/A RBD binds to gangliosides in the membrane. At acidic pH, the TD facilitates translocation of the LC across the membrane delivering the enzyme to the cytoplasm.^12^ The residual TD leaves a transmembrane pore once cleared of the LC.^13,14^

Because PFPs play so many biological roles and include potent human toxins, detection of pore-forming activity is essential for their identification and characterization.^16^ Several ensemble assays have been developed to detect PFPs by monitoring dequenching of fluorescent dyes from liposomes after pore formation.^17,18^ Ensemble measurements contain a heterogeneous mixture of intact and permeabilized liposomes and also suffer from low detection sensitivity. Many single molecule assays have been developed using liposomes.^19^ However, adaptation of dye leakage assays for single molecule imaging is problematic because it requires measurement of changes in fluorescence intensity.^20^ The intensity of dye-containing liposomes varies within a population so the leakage events must be captured in real time to observe the intensity change.^21,22^ An ideal single molecule sensor would report on the integrity of liposomes with a single measurement and could detect PFPs regardless of mechanism.

DNA oligonucleotides and aptamers are widely used as biosensors due to their ability to recognize ions, small molecules and even proteins.^23^ Often, molecular recognition in DNA is coupled to conformational changes in the DNA itself.^24^ As such, single molecule fluorescence has been widely used to detect conformational changes in DNA.^12^ Oligonucleotides have already been encapsulated in liposomes that were made permeable by pore forming toxins.^25,26^ Here, we tested the encapsulation of two DNA biosensors to develop ligand-specific single molecule FRET assays to monitor liposome integrity and detect the dissipation of transmembrane gradients of protons and potassium ions. We show that such biosensors can be used for the detection of PFPs and to gain insights regarding their mechanism of pore formation.

## Results

### Characterization of the Oligonucleotide Biosensors

We chose two biologically essential transmembrane gradients to test the utility of encapsulated oligonucleotides as biosensors: proton gradients and potassium gradients. We identified two ligand-responsive oligonucleotides with demonstrated sensitivity for each ion (Figure 2). For the proton sensor, we used a DNA sequence that is capable of forming an intramolecular triple-helix, in which a single-stranded cytosine–thymine (CT)-rich overhang folds back into the stem of a complementary duplex to form the triplex. Triplex DNA structures were first described in 1957 by Felsenfeld et al. and are stabilized by hydrogen bonds.^28^ In our case, we adapted a sequence from Brucale et al.,^27^ in which reversible triplex formation was demonstrated as a function of pH. The cytosine within CT-rich overhang must be protonated to hydrogen bond effectively with a G–C base pair within the stem to form stable C^+^•G–C base triads. The requirement for cytosine protonation makes this folding strongly pH-dependent, with triplex formation favored at acidic pH, and this folding behavior has already been adapted for single molecule fluorescence (Figure 2A).^27,28,31^ For the potassium sensor, we used human telomeric repeats, which protect the ends of chromosomes by folding into a G-quadruplex structure. This folding is strongly dependent on potassium and has also been characterized with single molecule FRET (Figure 2B).^29,30^ Thus, both oligonucleotides undergo biological conformational changes that depend on specific ions. To develop them as biosensors, we coupled the conformational change to produce a stable FRET signal that switched in response to their natural ligand ion. Both oligonucleotides were fluorescently labeled such that the folded structure showed high FRET efficiency while the unfolded state gave FRET efficiency near zero.

**Figure 2:**
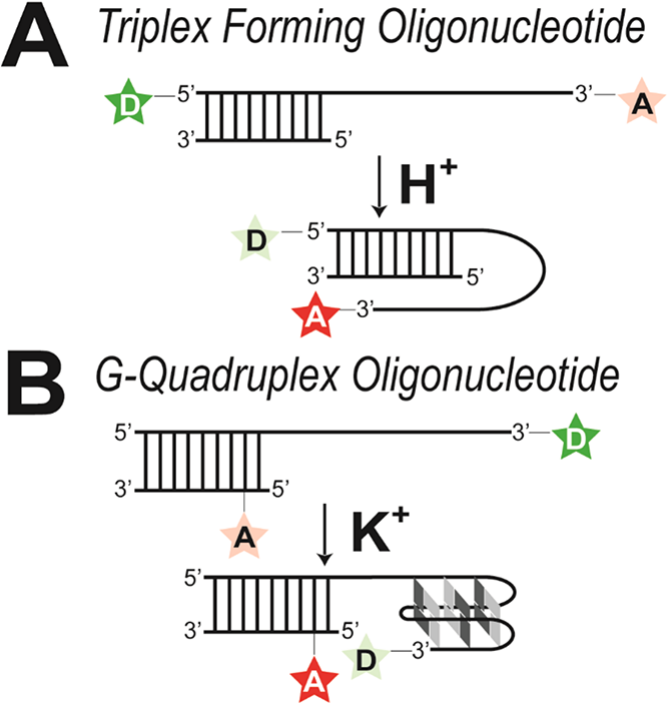
Structural transitions underlying ion detection by oligonucleotide biosensors. Oligonucleotides are represented schematically by lines. The sites of dye attachment are indicated for the donor (D, green star) and the acceptor (A, red star) with the color saturation indicating fluorescence emission. (A) The Triplex-Forming Oligonucleotide serves as a proton sensor.^27,28^ At neutral pH, the oligonucleotide remains in an extended conformation, spatially separating donor and acceptor. Upon proton binding, the overhang forms an intramolecular triple helix, bringing the donor and acceptor into proximity producing acceptor emission through Fluorescence Resonance Energy Transfer (FRET). (B) The G-Quadruplex Oligonucleotide serves as a potassium sensor.^29,30^ In lithium, the oligonucleotide remains in an extended conformation with the spatially separated dyes producing donor emission. Upon potassium binding, the overhang folds into a G-quadruplex structure, shown in the antiparallel basket conformation. This ligand induced conformational change brings the donor and acceptor into proximity, resulting in acceptor emission through FRET.

To benchmark the ion dependence of our labeling scheme for the oligonucleotides, we first measured single molecule FRET efficiency using total internal reflection microscopy (TIRF). We added biotin to the 3′ end of the shorter oligonucleotide and attached the DNA to a passivated microscope slide that had been sparsely coated with streptavidin (Figure 3A). We used alternating laser excitation^32^ to identify single molecules with active donor–acceptor pairs and measure FRET. For the proton sensor, in neutral buffer (25 mM sodium phosphate pH 8), the FRET histogram showed a single dominant population centered at raw FRET = 0.1, with 100% of molecules below raw FRET = 0.5; under acidic conditions (25 mM sodium phosphate pH 5.8), the distribution shifted dramatically, with a dominant population centered at raw FRET = 1.1 and >95% of molecules above FRET = 0.5 (Figure 3B). For the potassium sensor, the measurement in lithium-containing buffer (20 mM Tris, 100 mM LiCl, pH 7.5) yielded a single dominant population centered at raw FRET = −0.1, with 100% of molecules below raw FRET = 0.5; upon replacement with potassium (20 mM Tris, 100 mM KCl, pH 7.5), the FRET histogram shifted to a population centered at raw FRET = 1.0, with >95% of molecules above raw FRET = 0.5 (Figure 3C). Representative single molecule traces for both oligonucleotides showed stable FRET efficiency with no dynamics. The FRET transition for both sensors was so complete as to provide unambiguous detection of their target ligand. As such, the raw FRET efficiency was interpreted qualitatively as a binary response indicator.

**Figure 3:**
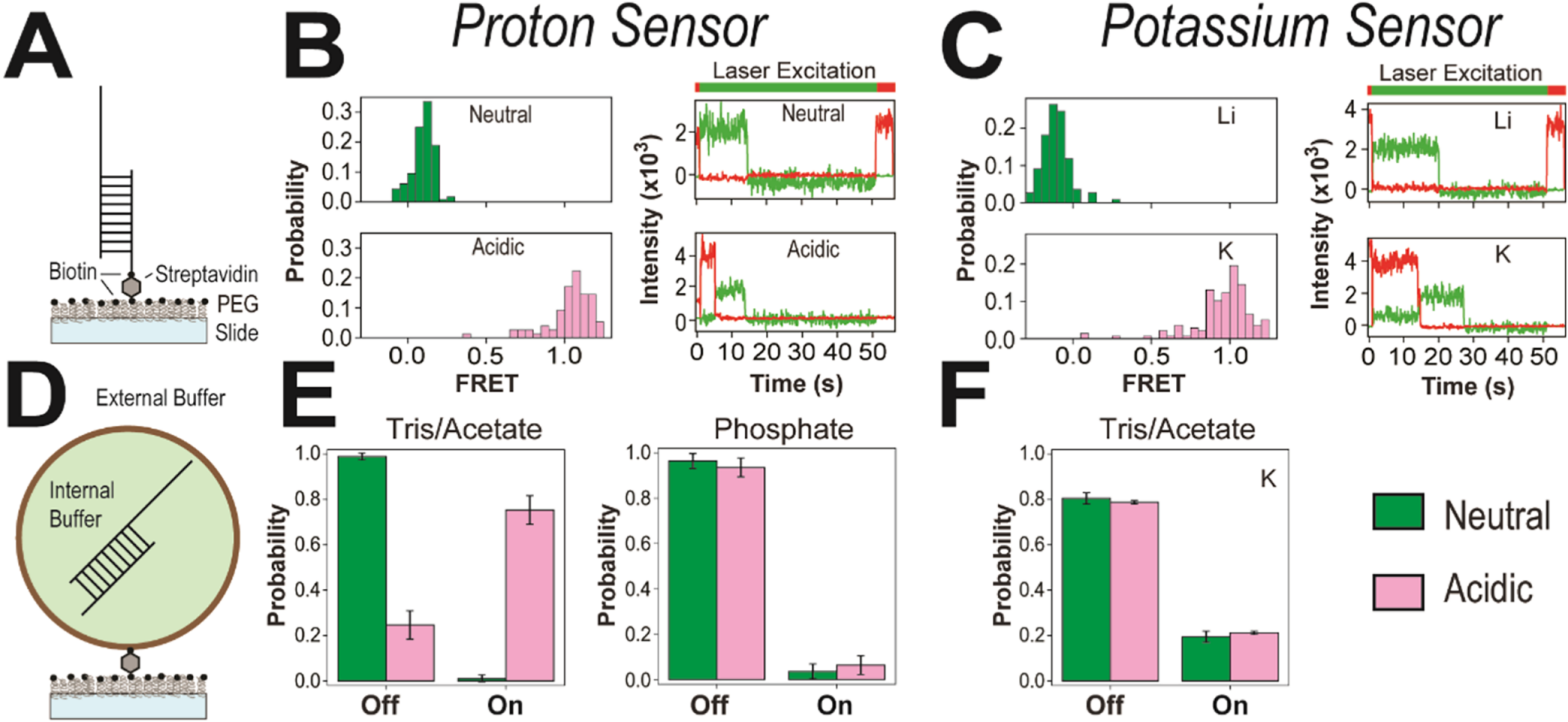
Effect of encapsulation on the oligonucleotide biosensors. (A) Schematic representation of the single-molecule FRET measurements using biotinylated oligonucleotides tethered to the slide surface via streptavidin. (B) Measurement of the surface attached proton sensor. *Left*: Population histograms showing the probability distributions of FRET values for the proton sensor in neutral buffer (green, *N* = 118) and acidic buffer (pink, *N* = 89). *Right*: Representative single molecule traces of donor (green) and acceptor (red) emission over time showing molecules in neutral buffer (top) or acidic buffer (bottom). (C) Measurement of the surface attached potassium sensor. *Left*: Population histograms showing the probability distributions of FRET values for the potassium sensor in neutral buffer containing lithium (green, *N* = 114) or potassium (pink, *N* = 166). *Right*: Representative single molecule traces of donor (green) and acceptor (red) emission over time, in lithium (top) and in potassium (bottom). (D) Schematic representation of single-molecule FRET measurements using oligonucleotides encapsulated within liposomes that are tethered to the slide surface via streptavidin. The internal buffer within liposomes is the original buffer from encapsulation. The external buffer can be exchanged to create chemical gradients. (E) Fraction of encapsulated proton sensor observed in the FRET-Off and FRET-On states in neutral buffer (green) and acidic buffer (pink). *Left*, in Tris/acetate buffers. *Right*, in phosphate buffers. (F) Fraction of encapsulated potassium sensor observed in the FRET-Off and FRET-On states in neutral Tris buffer (green) and acidic acetate buffer (pink) both supplemented with 100 mM KCl in the external buffer. A typical histogram contained ≥ 100 molecules. Three replicate histograms were generated for all bar plots. Histograms used in the analysis are shown in Figure S1.

Next we encapsulated the biosensors in 100 nm liposomes composed of phosphatidylcholine with 0.5% biotinylated phosphatidylethanolamine to allow individual liposomes to be attached to the passivated microscope slide.^33^ Oligonucleotides were encapsulated under neutral and potassium-free conditions. Because the liposome membrane is impermeable to charged molecules, the encapsulated oligonucleotides remain in the encapsulation buffer from when the liposome is formed unless permeabilized. Exchanging the external buffer allows us to create chemical gradients across the liposome membrane but in our experiments the osmolarity remains the same on both sides of the membrane (Figure 3D). The 100 nm liposome size was chosen to maintain physical stability while maximizing the probability of encapsulating a single DNA molecule per vesicle. The low internal volume of 100 nm liposomes naturally limits the number of encapsulated molecules, and under dilute loading conditions, the encapsulation process follows Poisson statistics, resulting in the majority of liposomes remaining empty. We estimated the encapsulation efficiency for the oligonucleotides to be 19 ± 3% by measuring colocalization of the acceptor-labeled oligonucleotide with a lipid marker. Although most liposomes do not contain DNA biosensors, this strategy ensures that singly loaded liposomes greatly outnumber multiply loaded ones, which is essential for clean, binary single-molecule FRET readouts. Moreover, the high surface density and wide imaging field in TIRF microscopy enable acquisition of hundreds of single-molecule traces per condition, providing sufficient throughput.

We first measured the FRET efficiency as with the surface-attached oligonucleotides in the absence of any pore-forming sources as a control. With the proton sensor, we found that encapsulated oligonucleotides maintained the FRET-Off state in neutral buffer (20 mM Tris, 100 mM NaCl, pH 7.5). However, we found that the use of acetate as the low pH buffer ion (20 mM sodium acetate, 100 mM NaCl, pH 5.5) lead to triggering of triple helix formation even in the absence of PFPs, with 75% of liposomes exhibiting FRET-On signals (Figure 3E). This suggests that protonated acetate, which is neutral, may be capable of crossing the membrane and disrupting the proton gradient. Maintenance of a transmembrane pH gradient was only possible when using 25 mM sodium phosphate as the sole buffer for both neutral and acidic conditions, because it remains negatively charged in both protonation states. The bar plot showed 96% and 94% of liposomes remained in the FRET-Off state in 25 mM sodium phosphate at pH 8 and pH 5.8 respectively (Figure 3E).

In contrast, encapsulation of the G-quadruplex oligonucleotide in liposomes resulted in a FRET-On population of ∼20% that was not seen in the surface measurements under either neutral or acidic conditions (Figure 3F). Examination of individual traces revealed that the high FRET population arose from a subset of molecules in a stable high FRET state rather than dynamic transitions. Thus, encapsulation biased the conformational distribution of the G-quadruplex but not the triple helix. To account for this, we subtracted the false positive rate of the potassium sensor-measured under control conditions without PFPs from the total positive probability to estimate the pore formation probability for the experimental conditions with PFPs.

### Single Liposome Detection of Pore Formation by Membrane Active Peptides

To test the sensitivity of our encapsulated biosensors for the dissipation of transmembrane gradients, we first examined the membrane active peptide GALA (Figure 1A).^7,8^ We encapsulated the proton sensor in phosphatidylcholine liposomes containing neutral phosphate buffer and measured the FRET efficiency distribution from surface attached liposomes. We repeated the measurements after incubating the liposomes in neutral or acidic phosphate buffer both in the absence (control) and presence of the GALA peptide at a 500:1 peptide:liposome ratio (Figure 4A). Across these conditions, we compared the probability of the biosensor being in a FRET-On state, which we equate to the Pore Formation Probability. Using the proton sensor, we observed a pore formation probability of 82% only when the GALA peptide was present under acidic conditions (Figure 4B). Similarly, we encapsulated the potassium sensor in neutral Tris buffer with LiCl and then incubated the liposomes with KCl in neutral Tris buffer or acidic acetate buffer both in the absence (control) and presence of the GALA peptide at a 500:1 peptide:liposome ratio. Using the potassium sensor, we similarly observed a *raw* pore formation probability of 95% only when the GALA peptide was present under acidic conditions (Figure 4C). Here, we did not apply the false-positive rate correction to the potassium sensor, as the goal was to validate the sensor’s ability for detecting pH-dependent PFP-triggered gradient dissipation, rather than comparing the two sensors. Our biosensor results are consistent with previous studies, which used ensemble dye leakage to show the pH dependence of pore formation by the GALA peptide.^8^

**Figure 4:**
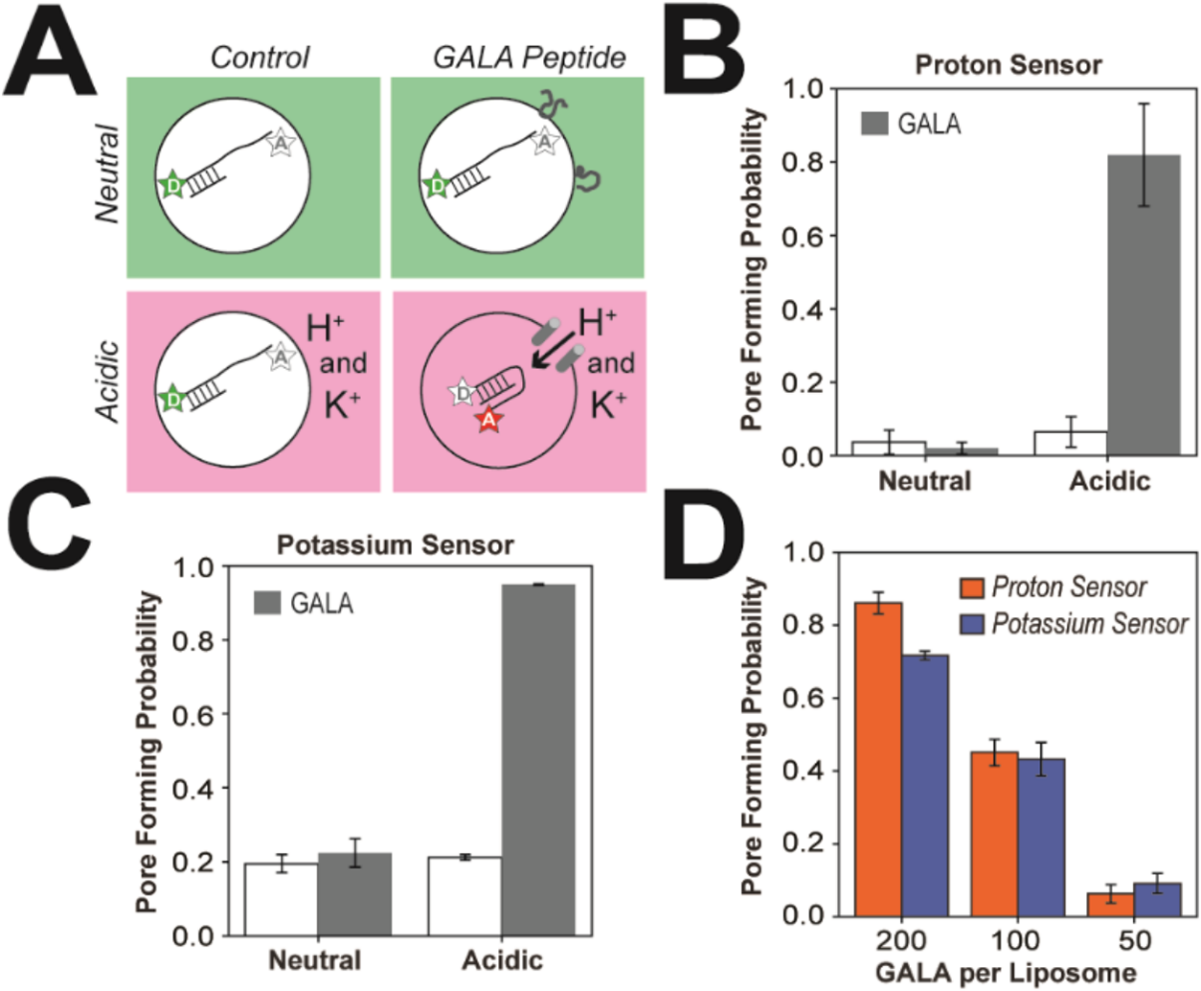
Detecting dissipation of transmembrane gradients by the membrane active GALA peptide. (A) Schematic of the four experimental conditions used to benchmark the pore sensors. As illustrated with the proton sensor and GALA, labeled oligonucleotides are shown encapsulated in the extended FRET-Off state. *Left*, control measurements of the encapsulated sensors under neutral (green) and acidic conditions (pink) lacking PFPs. *Right*, measurements of the encapsulated sensors under neutral (green) and acidic conditions (pink) in the presence of PFPs. Pore formation dissipates the transmembrane ion gradient triggering the FRET-On state. (B) Bar plots showing the pore-forming probability of the proton sensor under neutral and acidic conditions, in the absence (white) and presence (gray) of GALA. Histograms used in the analysis are shown in Figure S2. (C) Bar plots showing the pore-forming probability of the potassium sensor under neutral and acidic conditions supplemented with potassium, in the absence (white) and presence (gray) of GALA. Histograms used in the analysis are shown in Figure S2. (D) Bar plots comparing the pore-forming probability as a function of the GALA peptide to liposome ratio for the proton sensor (orange) and the potassium sensor (blue) under acidic conditions. Histograms used in the analysis are shown in Figure S3.

Next we examined the efficiency of pore formation by the GALA peptide by encapsulating the biosensors in liposomes composed of phosphatidylcholine and phosphatidylserine to mimic biological membranes. We incubated the liposomes under acidic conditions with GALA peptide at decreasing peptide:liposome ratios using phosphate buffer with the proton sensor and acetate buffer with KCl for the potassium sensor. We measured the pore forming probability using single molecule TIRF microscopy. At a 200:1 peptide:liposome ratio, we observed substantial pore formation events with an 86% and 72% pore-forming probability for the proton and potassium sensors respectively, but the probability decreased significantly at lower ratios. For both sensors, the probability was about 40% and 10% at a peptide:liposome ratio of 100:1 and 50:1 respectively (Figure 4D). Both biosensors showed a similar concentration dependence, suggesting that GALA pores pass protons and potassium with similar efficiency. With our biosensors, we were able to distinguish intact liposomes from those permeabilized by GALA pores while the stability of the FRET signal allowed the measurement of pore formation across the entire slide without the need to capture events in real time within a single field of view.

### Single Liposome Detection of Pore Formation by Botulinum Neurotoxin

Next we used our encapsulated biosensors to gain insight into the pore forming mechanism of Botulinum Neurotoxin A (BoNT/A). We used a catalytically inactive BoNT/Ai,^15,34^ which was expressed recombinantly and purified as a single polypeptide. To mimic the natural proteolytic activation of the holotoxin, we engineered a cleavage site for Tobacco Etch Virus (TEV) protease into the activation loop of the toxin. This loop is normally cleaved by host or bacterial proteases to convert the holotoxin into its functional, two-chain form composed of a heavy and light chain linked by a disulfide bond. The TEV site was introduced at a defined position to replace the native cleavage region while minimizing disruption to adjacent structural elements. TEV protease was chosen for its high sequence specificity, ensuring precise and efficient activation of the toxin. SDS-PAGE analysis confirmed complete and selective cleavage of BoNT/Ai following TEV treatment, yielding the expected heavy and light chain fragments (Figure S4). We examined the efficiency of pore formation by BoNT/Ai using liposomes composed of phosphatidylcholine and phosphatidylserine to mimic biological membranes. As with GALA, we incubated the liposomes under acidic conditions with BoNT/Ai at decreasing peptide:liposome ratios. We compared the single-chain holotoxin to the activated two-chain toxin (Figure 5A). The bar plots showed a clear increase in pore forming probability for the activated BoNT/Ai. At all peptide:liposome ratios tested, the two-chain BoNT/Ai yielded more than twice the pore forming probability of the holotoxin for both sensors. At the lowest ratio (5:1) where the holotoxin pore forming probability dropped to near zero and the gradient dissipations became barely detectable (1 and 2% for the proton and potassium sensors respectively), we still captured 24 and 9% of liposomes reporting pore formation for the proton and potassium sensors respectively with the activated BoNT/Ai. The higher probability for pore formation by the activated toxin compared to the holotoxin indicates that proteolytic activation promoted the translocation process and made pore formation more efficient.

**Figure 5:**
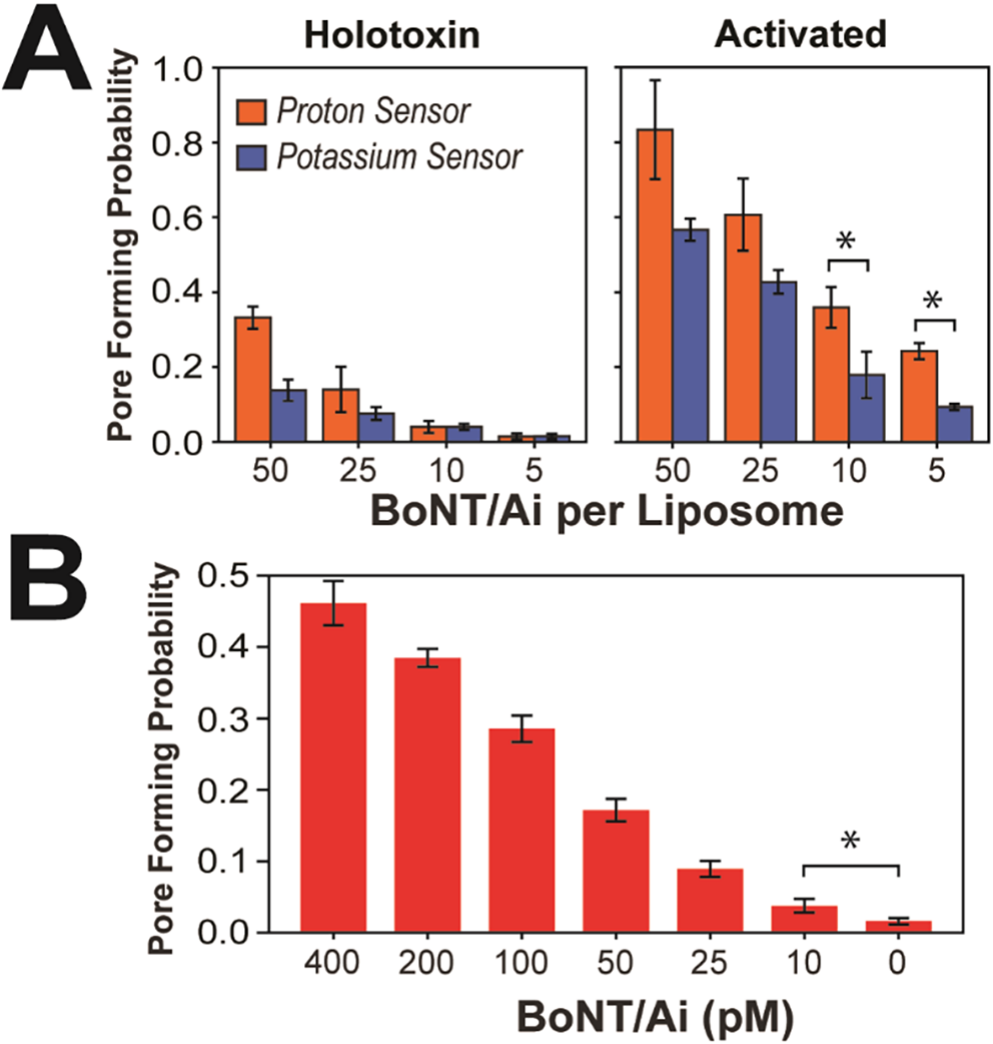
Detecting dissipation of transmembrane gradients by botulinum neurotoxin A (BoNT/Ai). (A) Bar plots comparing the pore-forming probability of the proton sensor (orange) and the potassium sensor (blue) as a function of the protein to liposome ratio of BoNT/Ai under acidic conditions. *Left*, Concentration dependence of the single-chain holotoxin. *Right*, Concentration dependence of the proteolytically activated two-chain toxin. The pore forming probability of the potassium sensor was corrected for false positives based on control experiments. Histograms used in the analysis are shown in Figure S5. (B) Detection threshold sensitivity for activated BoNT/Ai using the proton sensor encapsulated in GT1b-containing liposomes. Bar plots show the pore-forming probability for surface-attached liposomes triggered by exposure to the indicated concentrations of activated BoNT/Ai. Histograms used in the analysis are shown in Figure S6. * indicates significance at *p* < 0.05.

The activated BoNT/Ai also formed pores at much lower peptide:liposome ratios than the GALA peptide. When GALA was incubated at a 50:1 ratio, the pore forming probabilities of the proton and potassium sensors dropped to 6% and 9% respectively. Under the same incubation conditions, the activated BoNT/Ai permeabilized 83% of liposomes for the proton sensor and 57% for the potassium sensor and continued to be effective when the ratio used for incubations dropped to 5:1 (Figure 5A). This raised the question as to the sensitivity limit of our biosensor platform for the detection of activated BoNT/Ai.

We used only the proton sensor to investigate further due to the absence of false positives, which were seen with the potassium sensor. To mimic the biological membrane more closely, we incorporated the ganglioside sphingolipid GT1b, which acts as a physiological coreceptor for targeting BoNT/Ai through the receptor binding domain. We attached the liposomes to the passivated microscope slide surface and then exposed the surface-attached liposome array to acidic conditions (25 mM sodium phosphate pH 5.0) in the presentence of decreasing concentrations of the toxin (Figure 5B). The pore formation probability declined gradually with concentration, from 46% at 400 pM to 4% at 10 pM. Importantly, even at 10 pM, the signal remained significantly above that of control conditions with liposomes not being exposed to BoNT/Ai. This sensitivity approaches the efficacy of immunodetection without the need for protein-specific reagents.

## Discussion

Living systems require an asymmetric distribution of ions and nutrients to provide cellular energy and also permit controlled physiochemical conditions for biological reactions. These asymmetric distributions are enabled by biological phospholipid membranes, which restrict permeability. Nature has evolved a diverse set of transmembrane proteins that control the movement of ions and biomolecules across cellular membranes, often with high chemical specificity. In contrast, pore forming proteins create literal water-filled holes in the membrane that can nonspecifically dissipate transmembrane gradients. PFPs are a diverse class of polypeptides from across all living kingdoms that use different mechanisms to achieve the same effect. Detection of PFPs typically involves ensemble dye release assays or bespoke immunoreagents designed for specific PFP targets.

Here we report a surface-based assay to detect the dissipation of transmembrane gradients with single liposome resolution. The encapsulation of DNA oligonucleotides provides a robust system that functions across different buffer conditions. Using a stable FRET biosensor allowed liposomes to be interrogated after the reaction was complete, which eliminates the need to capture events in real time. Being able to interrogate hundreds of liposomes in a single measurement greatly increased the throughput needed to obtain statistical significance. While this study focused on detecting pore-forming activity, the platform is broadly adaptable. By selecting alternative ligand-responsive oligonucleotides, the system could be reconfigured to monitor other types of biomolecular activity that involve solute movement across membranes. For example, DNA-based sensors have been developed to respond to specific ions,^35^ small molecules such as ATP^36^ and glucose.^37^ Incorporating these sensors into liposomes would enable detection of ion channel gating, solute carrier transport, or pore formation induced by other pore-forming sources such as antimicrobial peptides or synthetic materials. Its single-liposome resolution also makes it well-suited for studying heterogeneous or transient membrane phenomena that are challenging to capture in ensemble assays.

We observed static FRET from both oligonucleotides, which allowed them to serve as binary response indicators for pore formation. Previous single-molecule FRET experiments on triple helix formation have reported dynamic folding transitions at neutral pH.^31^ However, these experiments used an unrelated oligonucleotide that contained a different internal linker and lower cytosine content.^38^ Additionally, single-molecule measurements of intramolecular quenching, instead of FRET, using the same oligonucleotides reported a complete duplex–triplex transition when switching between basic and acidic buffer conditions.^39^

Similarly, we found stable FRET with the potassium sensor while dynamics have been observed in G-quadruplex-forming sequences.^29,30^ Previous single molecule FRET studies using the same G-quadruplex-forming sequence reported dynamic interconversion between the chair and basket conformations but only in the presence of NaCl, which was not used here. This highlights the specificity of oligonucleotides in molecular recognition. Additionally, we observed high FRET from the encapsulated potassium sensor in LiCl that were not observed in solution, suggesting that lipid encapsulation alters the free energy landscape for G-quadruplex folding. That lipids could alter the energy landscape for the G-quadruplex is supported by the finding that chemical lipidation impacts G-quadruplex folding.^40,41^ Additionally, G-quadruplex sequences have been shown to facilitate membrane binding^42^ so it is possible that some encapsulated oligonucleotides bind the membrane with impacts on folding. The encapsulation-induced folding resulted in a false positive rate for the potassium sensor that we corrected with simple subtraction, but that prevents the potassium sensor from ever accurately reporting 100% leakage.

The encapsulation efficiency of oligonucleotides was relatively low (∼19%) in our experiments, but this outcome was expected based on Poisson loading statistics to avoid multiply loaded liposomes. We encapsulated the oligonucleotides under dilute conditions to maximize the single-molecule occupancy of liposomes. This ensured that we could examine single dye pairs to characterize the FRET signal. This trade-off in encapsulation efficiency results in only about a 5-fold reduction in throughput, since empty liposomes do not produce any fluorescent signal and are simply excluded from analysis. As a result, encapsulation efficiency does not compromise data quality or accuracy. We also note that multiply loaded liposomes are possible and may still provide a binary readout, as the presence of any FRET signal indicates that gradient dissipation has occurred, regardless of the number of encapsulated DNA oligonucleotide sensors. In the future, it may be possible to improve the encapsulation efficiency by using excess labeled oligonucleotides, as the FRET-based detection should not require having exactly one sensor per liposome. This flexibility could further enhance throughput without compromising interpretability of results.

Our biosensor measurements of GALA activity agreed well with the original characterization of the membrane active peptide.^7^ Our potassium sensor detected similar pore formation at pH 5.5, with both assays reaching about half-maximal leakage (43–60%) at 100 GALA/liposome. However, our proton sensor showed significantly higher sensitivity to pore formation with 82% at pH 5.8 while dye leakage was below 50% above pH 5.7 with 500 GALA/liposome.^7,8^ Thus, the stronger dependence of dye leakage on pH relative to ion leakage may arise from the large molecular weight and net negative charge of the dye rather than the efficiency of GALA pore formation at least for proton movement.

BoNT/A is known to form conducting channels.^13,14^ Single channel electrical recordings suggested that the channel is occluded by the light chain and requires proteolytic activation and reduction of the interchain disulfide bond to fully release channel blockage.^12^ Our pore sensors support a requirement for proteolytic activation to dissipate the transmembrane gradient. However, we did not reduce the intrachain disulfide bond so it seems likely that pore formation was incomplete. BoNT/A channel formation proceeds through stages of increasing conductance.^43^ Thus, our results suggest that even the low conductance states are sufficient to dissipate liposome gradients with varying degrees of efficiency. Interestingly, the BoNT/Ai pores were significantly more likely to release the proton gradients than the potassium gradients at all concentrations (Figure 5B), suggesting selective permeability of the occluded or partially formed channel. This may result from both ion-specific properties and pore structural features. Protons exist primarily as hydronium ions (H_3_O^+^) and can traverse narrow or occluded pores via the Grotthuss mechanism,^44,45^ while potassium ions, with their larger size and stable hydration shell, are less compatible with confined or partially formed channels. Structurally, light chain occlusion likely limits pore diameter, but may permit transient water wires or small-scale fluctuations that allow proton leakage. Moreover, ionizable residues within the transmembrane domain might favor proton passage over bulkier monovalent cations. Our control experiments (Figure 3E) revealed that protons can use acetate ions to cross the bilayer by neutralizing the net charge through protonation. Together, these results suggest that low-conductance BoNT/Ai pore states are sufficient to dissipate proton gradients, and that both ion properties and pore architecture contribute to the observed selectivity. It may be that reduction of the intrachain disulfide would further enhance the pore formation probability. This finding highlights how single liposome detection of transmembrane solute movement can facilitate mechanistic understanding of transport pathways.

## Methods

### DNA Oligonucleotides

All the DNA oligonucleotides used in this study were purchased from Integrated DNA Technologies. For the proton sensor, the triplex-forming strand sequence was 5′-CTC TCT CCT TTC TCC TGT ACA TCC TCT TTC CTC TC-3′,^27^ which was labeled with Cy3 at the 5′ end and labeled with Atto647 at the 3′ end. The complementary strand sequence was 5′-AGG AGA AAG GAG AGA G-3′. For the potassium sensor, the G-quadruplex-forming strand sequence was 5′-GCA GGC GTG GCA CCG GTA ATA GGA TTA GGG TTA GGG TTA GGG TTA GGG TTA GGG-3′, labeled with Cy3 at the 3′ end. The complementary stem strand sequence was 5′- AAC CCX AAT CCT ATT ACC GGT GCC ACG CCT GC - 3′, where X denotes an amino-C6-dT base for NHS ester labeling.^29^ Atto647 dye was conjugated to the labeling site at a 10:1 dye-to-oligonucleotide ratio in 0.1 M NaHCO_3_ at room temperature for 1 h. The reaction was quenched with Tris, and the labeled stem oligonucleotide was purified by ethanol precipitation using Oligo Clean & Concentrator Kits (Zymo Research) to remove free dye. For surface immobilization, biotinylated versions of both sensors included biotin attached to the 3′ end of the shorter strand. Both sensors were annealed by mixing an equimolar ratio of complementary DNA oligonucleotides at a concentration of 2.5 μM and heating to 95 °C for 5 min, followed by gradual cooling to room temperature over a few hours. The proton sensor was annealed in 100 mM phosphate, 1 mM MgCl_2_, pH 7.5, while the potassium sensor was annealed in 20 mM Tris, 100 mM LiCl, pH 7.5.

### Liposome Encapsulation

Phospholipids were purchased from Avanti Research. Three different lipid compositions were used to test the sensors and mimic biological membranes: 100% egg phosphatidylcholine (EPC), a mixture of 78% EPC and 22% brain phosphatidylserine (BPS), and a mixture of 70% EPC, 20% BPS, and 10% ganglioside GT1b (Santa Cruz Biotechnology). Each composition included 0.5% biotinylated Phosphatidylethanolamine (biotin-PE) for surface immobilization. The lipid mixture was initially dried from chloroform under argon gas flow using rotary evaporation to a thin lipid film, which was then placed in a vacuum desiccator for 1 h to remove any residual chloroform. The dried lipid was resuspended by vortexing followed by 5 freeze–thaw cycles in liquid nitrogen and room temperature water. The biosensors were encapsulated by extrusion by adding 0.5 μM of the annealed oligonucleotides to the hydrated lipids at a final concentration of 20 mg/mL. To achieve encapsulation and produce uniform small unilamellar vesicles (SUV), the lipid-DNA mixture was extruded 20 times through a 100 nm membrane filter using a mini extruder (Avanti Research). The extruded sample was desalted using a Sepharose CL-4B (Cytiva) to remove any unencapsulated oligonucleotides. We measured the encapsulation efficiency to be 19 ± 3% by using acceptor-only oligonucleotides encapsulated in rhodamine-labeled liposomes.

### Preparation of Pore Forming Proteins

The GALA peptide was synthesized by Genscript. The catalytically dead BoNT/Ai holotoxin was expressed in *E. coli* BL21 Star (DE3) (Invitrogen) as an N-terminal GST fusion protein.^15^ We introduced a Tobacco Etch Virus (TEV) protease site into the activation loop through site-directed mutagenesis (Genscript) (Figure S4A). After cell lysis by sonication, the lysate was clarified by centrifugation, and the soluble fraction was incubated with Glutathione Sepharose 4B beads (Thermo Scientific Pierce). After washing, the protein was eluted by on-column cleavage with PreScission protease. For BoNT/Ai activation, TEV protease was used to cleave the linker, generating a two-chain toxin with the heavy and light chains still connected by the disulfide bond (Figure S4B). Both the single chain holotoxin and the activated two-chain toxin were further purified using size exclusion chromatography on Superdex 200 (Cytiva) and stored in 20 mM Hepes, 150 mM NaCl, pH 7.4.

### Slide Passivation

Quartz microscope slides were cleaned by sequential sonication in acetone, ethanol and potassium hydroxide followed by oxygen plasma cleaning (Harrick Plasma).^46^ The slide surface was passivated in a single step using methoxy-polyethylene glycol (mPEG)-silane (Laysan Bio) with 1% molar ratio of biotin-polyethylene glycol (PEG)-silane (Laysan Bio) to permit surface attachment. Slides were rehydrated in water and buffer before incubation with streptavidin (Invitrogen). To prevent nonspecific binding, a mixture of Biolipidure 203 and Biolipidure 206 (NOF AMERICA Corporation) was applied before sample deposition. Biotinylated oligonucleotides or liposomes were then incubated in buffer to achieve the deposition of an optically resolved field of single-molecules.

### Experimental Incubation Conditions

For the potassium sensor, the neutral buffer was 20 mM Tris pH 7.5 while the acidic buffer was 20 mM potassium acetate pH 5.5. These were supplemented with either 100 mM LiCl or 100 mM KCl as indicated. For the proton sensor, we initially tried 20 mM Tris pH 7.5 with 20 mM sodium acetate pH 5.5 supplemented with 100 mM NaCl. However, encapsulated control experiments showed that acetate dissipated the transmembrane pH gradient. Thus, all experiments were conducted in 25 mM phosphate at either pH 8 for neutral conditions or pH 5.8 for acidic conditions, which sustained the transmembrane pH gradient. PFPs at the indicated protein-to-liposome ratios were incubated with liposomes (25 μM lipid concentration) in microfuge tubes at 25 °C for 5 min, prior to being diluted and attached to the surface. To measure our detection limit for BoNT/Ai, incubations were performed directly on slides in 25 mM phosphate, pH 5. The liposomes containing encapsulated proton sensor were attached to the passivated surface and incubated with the indicated concentrations of activated BoNT/Ai at 25 °C, for 5 min before measurements.

### Single Molecule Microscopy

Images were acquired using a prism-based Total Internal Reflection Fluorescence (TIRF) microscope constructed on an IX71 Olympus base with a 60×/1.2-NA water-immersion objective. Imaging buffers matched the incubation buffers but included 1 mM trolox as a triplet state quencher. Oxygen scavenging was not used to aid bleaching of both donor and acceptor as a confirmation of a single dye pair. Laser excitation was filtered by a 550 nm long pass filter (Chroma). Fluorescence emission was spectrally separated using an Optosplit Image splitter (Cairn Research) containing a 650 nm dichroic mirror (Semrock) with a 550/100 nm bandpass filter (Chroma) for the donor and a 700/75 nm bandpass filter for the acceptor (Chroma). The spectrally resolved images were relayed onto separate halves of an iXon DU-897 EMCCD camera (Andor Technologies) and collected at a 10 Hz frame rate. We used alternating-laser excitation (ALEX) to sequentially excite the dyes.^32^ A 637 nm laser (Coherent Inc.) was initially used for 10 frames to identify active acceptors. Then a 532 nm laser (Laser Quantum) was used for 500 frames to identify active donors, capture energy transfer events and photobleach the dyes. Lastly, 637 nm excitation was used for 50 frames to confirm acceptor bleaching during FRET excitation.

### Single Molecule Trace Analysis

Microscopy images were analyzed using MATLAB to correlate donor and acceptor images, extract single molecule raw intensity time traces as previously described.^47^ Python scripts were used to calculate the single molecule averaged FRET value from the raw intensity of donor and acceptor as

1 $$\mathrm{FRET}=\frac{I_{A}}{I_{A}+I_{D}}$$

Given the two distinct peaks in the FRET histograms, we classified the single molecule traces as a binary system using FRET = 0.5 as a cutoff value to assign molecules as either FRET-On (FRET > 0.5) or FRET-Off (FRET < 0.5). FRET-On traces were classified as permeabilized liposomes. The pore forming probability was calculated as

2 $$P_{pore\;for\min g}=\frac{\#\;of\;liposomes\;with\;formed\;pores}{\#of\;all\;liposomes}$$

The pore forming probability for the potassium sensor was corrected by subtracting the false positive rate determined from control experiments.

## Supplementary Material



## Notes

Population histograms of FRET from replicate measurements used for generating bar plots; experimental details for TEV cleavage site design and SDS-PAGE analysis confirming BoNT/Ai activation; tables summarizing FRET-On probability data from replicate measurements and statistical analysis of significance of experimental findings (PDF)

## References

[1] Gumz M. L., Rabinowitz L., Wingo C. S. (2015). An Integrated View of Potassium Homeostasis. N Engl J. Med..

[2] Stautz J., Hellmich Y., Fuss M. F., Silberberg J. M., Devlin J. R., Stockbridge R. B., Hänelt I. (2021). Molecular
Mechanisms for Bacterial Potassium Homeostasis. J. Mol. Biol..

[3] Maxfield F. R., McGraw T. E. (2004). Endocytic Recycling. Nat. Rev.
Mol. Cell Biol..

[4] Margheritis E., Kappelhoff S., Cosentino K. (2023). Pore-Forming Proteins: From Pore
Assembly to Structure by Quantitative Single-Molecule Imaging. Int. J. Mol. Sci..

[5] Mesa-Galloso H., Pedrera L., Ros U. (2021). Pore-Forming
Proteins: From Defense
Factors to Endogenous Executors of Cell Death. Chem. Phys. Lipids.

[6] Benton J. T., Bayly-Jones C. (2021). Challenges
and Approaches to Studying Pore-Forming
Proteins. Biochem. Soc. Trans..

[7] Subbarao N.
K., Parente R. A., Szoka F. C., Nadasdi L., Pongracz K. (1987). The Ph-Dependent
Bilayer Destabilization by an Amphipathic
Peptide. Biochemistry.

[8] Parente R. A., Nir S., Szoka F. C. (1990). Mechanism of Leakage of Phospholipid
Vesicle Contents Induced by the Peptide Gala. Biochemistry.

[9] Dong M., Masuyer G., Stenmark P. (2019). Botulinum and Tetanus Neurotoxins. Annu. Rev. Biochem..

[10] DasGupta B. R., Sugiyama H. (1972). A Common Subunit Structure
in Clostridium Botulinum
Type a, B and E Toxins. Biochem. Biophys. Res.
Commun..

[11] Lacy D. B., Tepp W., Cohen A. C., DasGupta B. R., Stevens R. C. (1998). Crystal
Structure of Botulinum Neurotoxin Type a and Implications for Toxicity. Nat. Struct. Biol..

[12] Koriazova L. K., Montal M. (2003). Translocation of Botulinum
Neurotoxin Light Chain Protease
through the Heavy Chain Channel. Nat. Struct.
Biol..

[13] Hoch D. H., Romero-Mira M., Ehrlich B. E., Finkelstein A., DasGupta B. R., Simpson L. L. (1985). Channels Formed by Botulinum, Tetanus,
and Diphtheria Toxins in Planar Lipid Bilayers: Relevance to Translocation
of Proteins across Membranes. Proc. Natl. Acad.
Sci. U.S.A..

[14] Donovan J. J., Middlebrook J. L. (1986). Ion-Conducting Channels Produced by Botulinum Toxin
in Planar Lipid Membranes. Biochemistry.

[15] Gu S., Rumpel S., Zhou J., Strotmeier J., Bigalke H., Perry K., Shoemaker C. B., Rummel A., Jin R. (2012). Botulinum Neurotoxin
Is Shielded
by Ntnha in an Interlocked Complex. Science.

[16] Ros U., Pedrera L., Garcia-Saez A. J. (2021). Techniques for Studying Membrane
Pores. Curr. Opin. Struct. Biol..

[17] Kendall D. A., MacDonald R. C. (1982). A Fluorescence
Assay to Monitor Vesicle Fusion and
Lysis. J. Biol. Chem..

[18] Ellens H., Bentz J., Szoka F. C. (1984). Ph-Induced
Destabilization of Phosphatidylethanolamine-Containing
Liposomes: Role of Bilayer Contact. Biochemistry.

[19] Christensen S. M., Stamou D. G. (2010). Sensing-Applications of Surface-Based Single Vesicle
Arrays. Sensors.

[20] Apellániz B., Nieva J. L., Schwille P., García-Sáez A. J. (2010). All-or-None
Versus Graded: Single-Vesicle Analysis Reveals Lipid Composition Effects
on Membrane Permeabilization. Biophys. J..

[21] Bowen M. E., Weninger K., Brunger A. T., Chu S. (2004). Single Molecule Observation
of Liposome-Bilayer Fusion Thermally Induced by Soluble N-Ethyl Maleimide
Sensitive-Factor Attachment Protein Receptors (Snares). Biophys. J..

[22] McGuinness C., Walsh J. C., Bayly-Jones C., Dunstone M. A., Christie M. P., Morton C. J., Parker M. W., Böcking T. (2022). Single-Molecule
Analysis of the Entire Perfringolysin O Pore Formation Pathway. eLife.

[23] Weng R., Lou S., Li L., Zhang Y., Qiu J., Su X., Qian Y., Walter N. G. (2019). Single-Molecule Kinetic Fingerprinting
for the Ultrasensitive Detection of Small Molecules with Aptasensors. Anal. Chem..

[24] Ellington A. D., Szostak J. W. (1992). Selection in Vitro
of Single-Stranded DNA Molecules
That Fold into Specific Ligand-Binding Structures. Nature.

[25] Okumus B., Wilson T. J., Lilley D. M., Ha T. (2004). Vesicle Encapsulation
Studies Reveal That Single Molecule Ribozyme Heterogeneities Are Intrinsic. Biophys. J..

[26] Nickel W., Weber T., McNew J. A., Parlati F., Söllner T. H., Rothman J. E. (1999). Content Mixing and
Membrane Integrity During Membrane
Fusion Driven by Pairing of Isolated V-Snares and T-Snares. Proc. Natl. Acad. Sci. U.S.A..

[27] Brucale M., Zuccheri G., Samorì B. (2005). The Dynamic
Properties of an Intramolecular
Transition from DNA Duplex to Cytosine-Thymine Motif Triplex. Org. Biomol Chem..

[28] Felsenfeld G., Davies D. R., Rich A. (1957). Formation
of a Three-Stranded Polynucleotide
Molecule. J. Am. Chem. Soc..

[29] Noer S. L., Preus S., Gudnason D., Aznauryan M., Mergny J. L., Birkedal V. (2016). Folding Dynamics and
Conformational
Heterogeneity of Human Telomeric G-Quadruplex Structures in Na+ Solutions
by Single Molecule Fret Microscopy. Nucleic
Acids Res..

[30] Tippana R., Xiao W., Myong S. (2014). G-Quadruplex Conformation and Dynamics
Are Determined by Loop Length and Sequence. Nucleic Acids Res..

[31] Lee I. B., Lee J. Y., Lee N.-K., Hong S.-C. (2012). Direct Observation
of the Formation of DNA Triplexes by Single-Molecule Fret Measurements. Curr. Appl. Phys..

[32] Kapanidis A. N., Laurence T. A., Lee N. K., Margeat E., Kong X., Weiss S. (2005). Alternating-Laser Excitation
of Single Molecules. Acc. Chem. Res..

[33] Boukobza E., Sonnenfeld A., Haran G. (2001). Immobilization in Surface-Tethered
Lipid Vesicles as a New Tool for Single Biomolecule Spectroscopy. J. Phys. Chem. B.

[34] Binz T., Bade S., Rummel A., Kollewe A., Alves J. (2002). Arg­(362) and
Tyr(365) of the Botulinum Neurotoxin Type a Light Chain Are Involved
in Transition State Stabilization. Biochemistry.

[35] Fu T., Ren S., Gong L., Meng H., Cui L., Kong R. M., Zhang X. B., Tan W. (2016). A Label-Free Dnazyme Fluorescence
Biosensor for Amplified Detection of Pb­(2+)-Based on Cleavage-Induced
G-Quadruplex Formation. Talanta.

[36] Nutiu R., Li Y. (2003). Structure-Switching
Signaling Aptamers. J.
Am. Chem. Soc..

[37] Xiang Y., Lu Y. (2011). Using Personal Glucose
Meters and Functional DNA Sensors to Quantify
a Variety of Analytical Targets. Nat. Chem..

[38] Ohmichi T., Kawamoto Y., Wu P., Miyoshi D., Karimata H., Sugimoto N. (2005). DNA-Based Biosensor
for Monitoring Ph in Vitro and
in Living Cells. Biochemistry.

[39] Kolaric B., Sliwa M., Brucale M., Vallée R. A. L., Zuccheri G., Samori B., Hofkens J., De Schryver F. C. (2007). Single
Molecule Fluorescence Spectroscopy of Ph Sensitive Oligonucleotide
Switches. Photochem. Photobiol. Sci..

[40] Swiatkowska A., Juskowiak B. (2014). Effect of
Cholesterol Anchoring Group on the Properties
of G-Quadruplex-Based Fret Probes for Potassium Ion. Chemosensors.

[41] Vialet B., Gissot A., Delzor R., Barthélémy P. (2017). Controlling
G-Quadruplex Formation Via Lipid Modification of Oligonucleotide Sequences. Chem. Commun..

[42] Czerniak T., Saenz J. P. (2022). Lipid Membranes
Modulate the Activity of Rna through
Sequence-Dependent Interactions. Proc. Natl.
Acad. Sci. U.S.A..

[43] Fischer A., Montal M. (2007). Single Molecule Detection
of Intermediates During Botulinum
Neurotoxin Translocation across Membranes. Proc.
Natl. Acad. Sci. U.S.A..

[44] Nagle J. F., Morowitz H. J. (1978). Molecular Mechanisms
for Proton Transport in Membranes. Proc. Natl.
Acad. Sci. U.S.A..

[45] Brzezinski P., Ädelroth P. (2006). Design Principles of Proton-Pumping Haem-Copper Oxidases. Curr. Opin. Struct. Biol..

[46] Choi U. B., Weninger K. R., Bowen M. E. (2012). Immobilization
of Proteins for Single-Molecule
Fluorescence Resonance Energy Transfer Measurements of Conformation
and Dynamics. Methods Mol. Biol..

[47] Hamilton G. L., Saikia N., Basak S., Welcome F. S., Wu F., Kubiak J., Zhang C., Hao Y., Seidel C. A. M., Ding F. (2022). Fuzzy Supertertiary
Interactions within Psd-95
Enable Ligand Binding. eLife.

