# Supplementary material for: A Biosensor Platform for Detecting the Dissipation of Transmembrane Gradients in Single Liposomes
Source: Anal Chem. 2025 Sep 19;97(38):20825–33. doi: 10.1021/acs.analchem.5c02841 (PMC12489898; doi:10.1021/acs.analchem.5c02841)
Supplement: Supplementary file 1 [file ac5c02841_si_001.pdf]

**Supporting Information**

# A Biosensor Platform for Detecting The Dissipation of Transmembrane Gradients in Single Liposomes.

Changcheng Zhang[1] and Mark E. Bowen[2*]

[1]Department of Physics & Astronomy Stony Brook University, Stony Brook, NY, 11794, USA

[2]Department of Physiology & Biophysics, Stony Brook University, Stony Brook, NY, 11794, USA

* To whom correspondence should be addressed. mark.bowen@stonybrook.edu

**Table of Contents**

Figure S1. Effect of Encapsulation on the Oligonucleotide Biosensors. S3

Figure S2. Detecting Dissipation of Transmembrane Gradients by the Membrane Active GALA peptide. S4

Figure S3. GALA Concentration Dependency for Triggering the Sensors. S5

Figure S4. Improved Proteolytic Activation of Botulinum Neurotoxin A. S6

Figure S5. BoNT/Ai Concentration Dependency for Triggering the Sensors. S7

Figure S6. BoNT/Ai Detection Limit with GT1b-containing Lipid Encapsulated Proton Sensor. S8

Table S1. FRET-On Probabilities and Statistical Summary for Encapsulated Biosensors under Control Conditions. S9

Table S2. FRET-On Probabilities and Statistical Summary for Encapsulated Biosensors in the Presence of GALA. S10

Table S3. FRET-On Probabilities and Statistical Summary for Encapsulated Biosensors in Response to Varying GALA: Liposome Ratios. S11

Table S4. FRET-On Probabilities and Statistical Summary for Encapsulated Biosensors in Response to Varying BoNT/Ai Holotoxin: Liposome Ratios. S12

Table S5. FRET-On Probabilities and Statistical Summary for Encapsulated Biosensors in Response to Varying Activated BoNT: Liposome Ratios. S13

Table S6. FRET-On Probabilities and Statistical Summary for the Encapsulated Proton Sensor in Response to Varying Activated BoNT/Ai Concentrations. S14

Supporting References S15

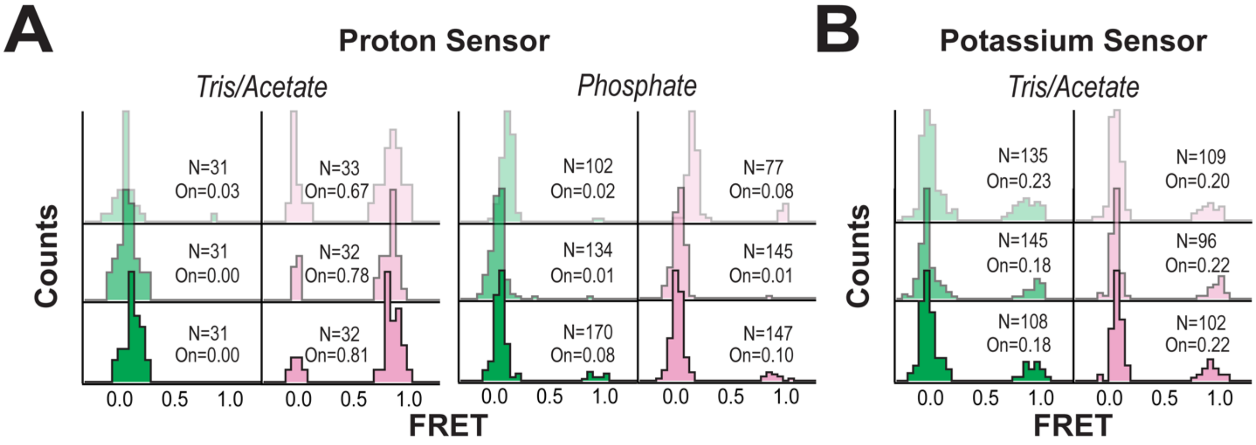


**Figure S1. Effect of Encapsulation on the Oligonucleotide Biosensors. A)** FRET histograms of the replicates used to generate the bar plot in Fig. 3E, showing the FRET distributions of the encapsulated proton sensor control experiments in neutral buffer (green) and acidic buffer (pink). Left, in 20 mM Tris, 100 mM NaCl, pH 7.5 and 20 mM sodium acetate, 100 mM NaCl, pH 5.5. Right, in 25 mM phosphate buffer, either at pH 8 or pH 5.8. **B)** FRET histograms of the replicates used to generate the bar plot in Fig. 3F, showing the FRET distributions of the encapsulated potassium sensor control experiments in 20 mM Tris, 100 mM KCl, pH 7.5 (neutral, green) and 20 mM potassium acetate, 100 mM KCl, pH 5.5 (acidic, pink). N indicates the number of molecules analyzed in each histogram; On value denotes the FRET-On fraction calculated for each histogram.

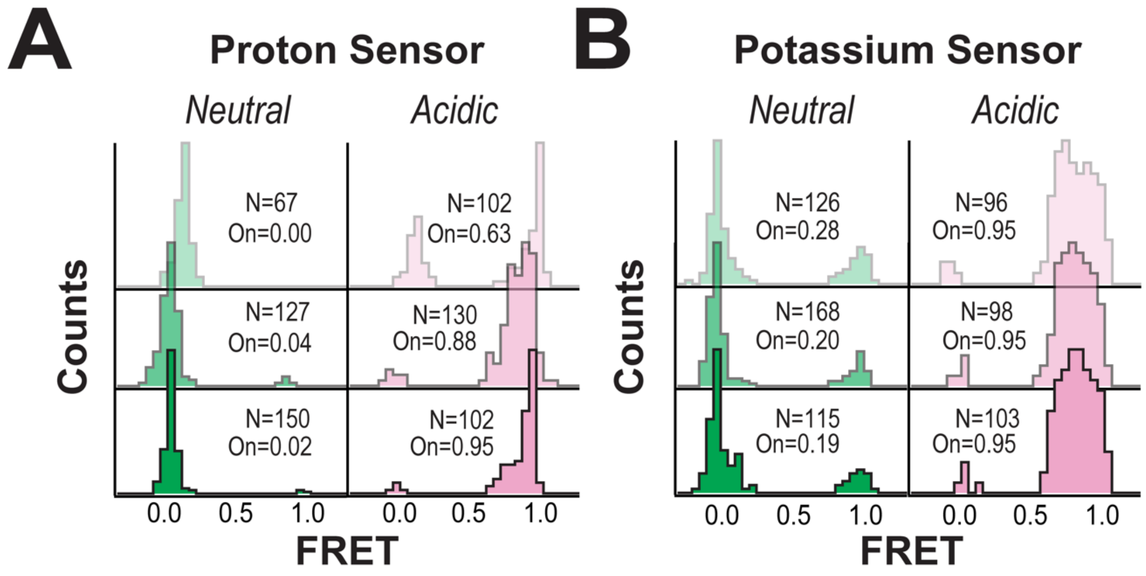


**Figure S2. Detecting Dissipation of Transmembrane Gradients by the Membrane Active GALA peptide. A)** FRET histograms of the replicates used to generate the bar plot in Fig. 4B, showing the FRET distributions of the encapsulated proton sensor observed in the presence of GALA, in 25 mM phosphate buffers, either pH 8 (neutral, green) or pH 5.8 (acidic, pink). **B)** FRET histograms of the replicates used to generate the bar plot in Fig. 4C, showing the FRET distributions of the encapsulated potassium sensor observed in the presence of GALA, in 20 mM Tris, 100 mM KCl, pH 7.5 (neutral, green) and 20 mM potassium acetate, 100 mM KCl, pH 5.5 (acidic, pink). N indicates the number of molecules analyzed in each histogram; On value denotes the FRET-On fraction calculated for each histogram.

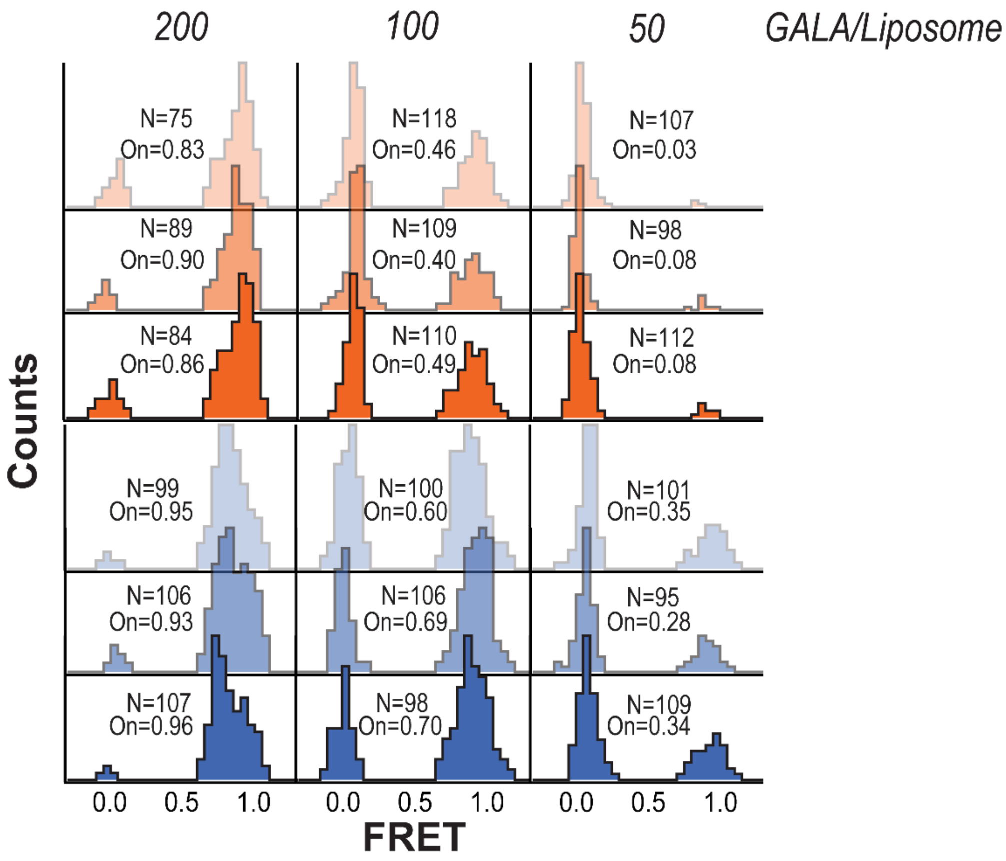


**Figure S3. GALA Concentration Dependency for Triggering the Sensors.** FRET histograms of the replicates used to generate the bar plot in Fig. 4D, showing the FRET distributions of the encapsulated proton sensor (orange) and the FRET distributions of the encapsulated potassium sensor (blue) observed at different GALA to liposome incubation ratios as indicated above in the panel. The proton sensor measurements were in 25 mM phosphate, pH 5.8, and the potassium sensor measurements were in 20 mM potassium acetate, 100 mM KCl, pH 5.5. N indicates the number of molecules analyzed in each histogram; On value denotes the FRET-On fraction calculated for each histogram.

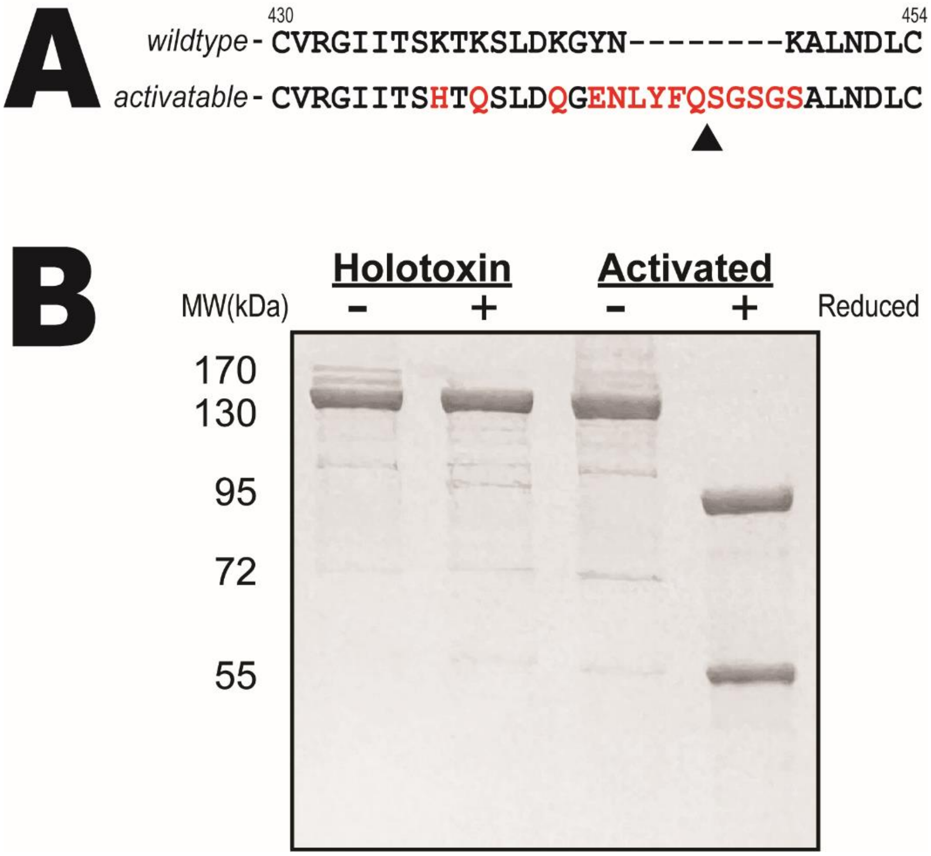


**Figure S4. Improved Proteolytic Activation of Botulinum Neurotoxin A.** BoNT/A is synthesized as a 149.5 kDa holotoxin, which is proteolytically cleaved by bacterial or host proteases into a 49.5 kDa light chain and an 98.1 kDa heavy chain. The two-chain toxin is held together by a disulfide bond. **A)** Engineering a Tobacco Etch Virus (TEV) cleave site into the activation loop to permit efficient and selective cleavage of the holotoxin. Shown is the amino acid sequence of the activation loop for wild type BoNT/A (*top*) and the mutations introduced to generate the activatable toxin (*bottom*). Site directed mutations are highlighted in red. Arrow indicates the TEV cleavage site. The natural cleavage sites were removed, as has been reported,[1] while GS repeats were added to achieve efficient cleavage. **B)** Efficiency of proteolytic activation by TEV protease. Shown is a Coomassie-stained Any kD™ gel (Bio-Rad) showing the single chain holotoxin (*left*) and the activated two-chain toxin produced by TEV cleavage (*right*). Samples were run without reducing agents (**-**) and with reducing agents (**+**), as indicated above each lane, to demonstrate the integrity of the disulfide bond in the samples used for experiments.

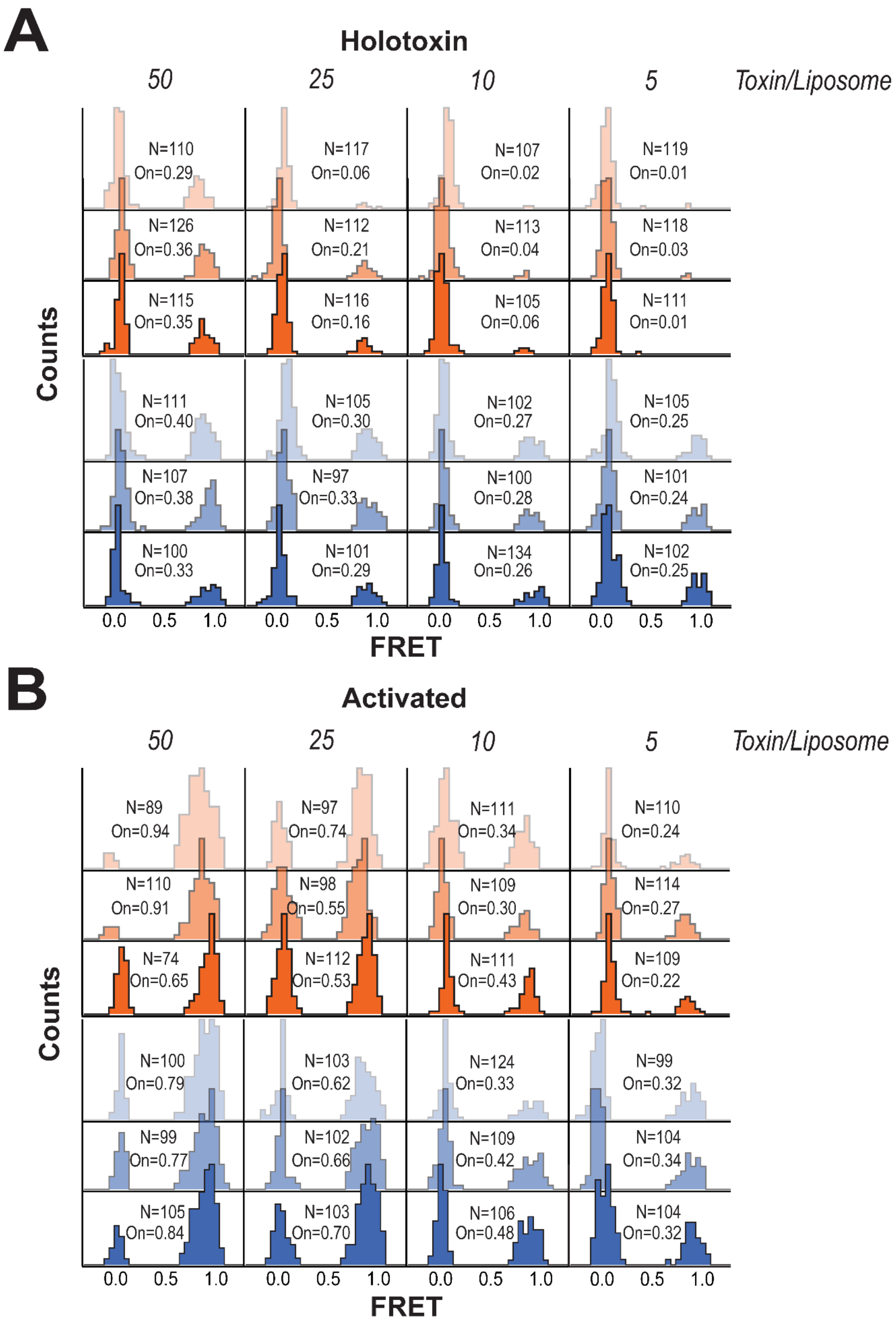


**Figure S5. BoNT/Ai Concentration Dependency for Triggering the Sensors.** FRET histograms of the replicates used to generate the bar plot in Fig. 5A, showing the FRET distributions of the encapsulated proton sensor (orange) and the FRET distributions of the encapsulated potassium sensor (blue) observed at different BoNT/Ai to liposome incubation ratios as indicated above in the panel, using **A)** single-chain holotoxin and **B)** proteolytic-activated two-chain toxin. The proton sensor measurements were in 25 mM phosphate, pH 5.8, and the potassium sensor measurements were in 20 mM potassium acetate, 100 mM KCl, pH 5.5. N indicates the number of molecules analyzed in each histogram; On value denotes the FRET-On fraction calculated for each histogram.

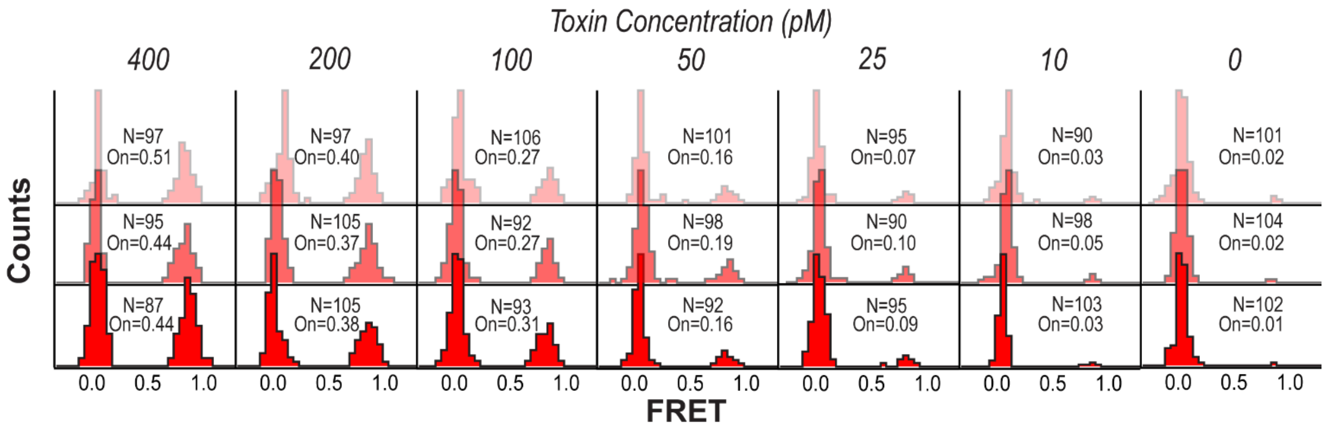


**Figure S6. BoNT/Ai Detection Limit with GT1b-containing Lipid Encapsulated Proton Sensor.** FRET histograms of the replicates used to generate the bar plot in Fig. 5B, showing the FRET distributions of the GT1b-containing lipid encapsulated proton sensor observed at different on-slide activated BoNT/Ai exposure concentrations as indicated above in the panel, in 25 mM phosphate, pH 5. N indicates the number of molecules analyzed in each histogram; On value denotes the FRET-On fraction calculated for each histogram.

**Table S1. FRET-On Probabilities and Statistical Summary for Encapsulated Biosensors under Control Conditions.**

The table presents FRET-On probability values from three independent experiments, along with the calculated average (Avg.) and standard deviation (S.D.) for both proton and potassium sensors under different buffer conditions corresponding to Figure S1.

| | Proton Sensor | | | | Potassium Sensor | |
|---|---|---|---|---|---|---|
| Buffer Condition | Tris pH 7.5 | Acetate pH 5.5 | Phosphate pH 8 | Phosphate pH 5.8 | Tris pH 7.5 | Acetate pH 5.5 |
| Exp. 1 | 0.03 | 0.67 | 0.02 | 0.08 | 0.23 | 0.20 |
| Exp. 2 | 0.00 | 0.78 | 0.01 | 0.01 | 0.18 | 0.22 |
| Exp. 3 | 0.00 | 0.81 | 0.08 | 0.10 | 0.18 | 0.22 |
| Avg. | 0.01 | 0.75 | 0.04 | 0.06 | 0.19 | 0.21 |
| S.D. | 0.02 | 0.06 | 0.03 | 0.04 | 0.02 | 0.01 |

**Table S2. FRET-On Probabilities and Statistical Summary for Encapsulated Biosensors in the Presence of GALA.**

The table presents FRET-On probability values from three independent experiments, along with the calculated average (Avg.) and standard deviation (S.D.) for both proton and potassium sensors under different buffer conditions corresponding to Figure S2.

| | Proton Sensor | | Potassium Sensor | |
|---|---|---|---|---|
| Buffer Condition | Phosphate pH 8 | Phosphate pH 5.8 | Tris pH 7.5 | Acetate pH 5.5 |
| Exp. 1 | 0.00 | 0.63 | 0.28 | 0.95 |
| Exp. 2 | 0.04 | 0.88 | 0.20 | 0.95 |
| Exp. 3 | 0.02 | 0.95 | 0.19 | 0.95 |
| Avg. | 0.02 | 0.82 | 0.22 | 0.95 |
| S.D. | 0.02 | 0.14 | 0.04 | 1.5E-3 |

**Table S3. FRET-On Probabilities and Statistical Summary for Encapsulated Biosensors in Response to Varying GALA: Liposome Ratios.**

The table presents FRET-On probability values from three independent experiments, along with the calculated average (Avg.) and standard deviation (S.D.) for both proton and potassium sensors under acidic conditions at a decreasing GALA to liposome ratio corresponding to Figure S3.

| | Proton Sensor | | | Potassium Sensor | | |
|---|---|---|---|---|---|---|
| GALA per Liposome | 200 | 100 | 50 | 200 | 100 | 50 |
| Exp. 1 | 0.83 | 0.46 | 0.03 | 0.95 | 0.60 | 0.35 |
| Exp. 2 | 0.90 | 0.40 | 0.08 | 0.93 | 0.69 | 0.28 |
| Exp. 3 | 0.86 | 0.49 | 0.08 | 0.96 | 0.70 | 0.34 |
| Avg. | 0.86 | 0.45 | 0.06 | 0.95 | 0.66 | 0.32 |
| S.D. | 0.03 | 0.04 | 0.02 | 0.01 | 0.05 | 0.03 |

**Table S4. FRET-On Probabilities and Statistical Summary for Encapsulated Biosensors in Response to Varying BoNT/Ai Holotoxin: Liposome Ratios.**

The table presents FRET-On probability values from three independent experiments, along with the calculated average (Avg.) and standard deviation (S.D.) for both proton and potassium sensors under acidic conditions at a decreasing single-chain BoNT/Ai holotoxin to liposome ratio corresponding to Figure S5A.

| | Proton Sensor | | | | | Potassium Sensor | | |
|---|---|---|---|---|---|---|---|---|
| BoNT per Liposome | 50 | 25 | 10 | 5 | 50 | 25 | 10 | 5 |
| Exp. 1 | 0.29 | 0.06 | 0.02 | 0.01 | 0.40 | 0.30 | 0.27 | 0.25 |
| Exp. 2 | 0.36 | 0.21 | 0.04 | 0.03 | 0.38 | 0.33 | 0.28 | 0.24 |
| Exp. 3 | 0.35 | 0.16 | 0.06 | 0.01 | 0.33 | 0.29 | 0.26 | 0.25 |
| Avg. | 0.33 | 0.14 | 0.04 | 0.01 | 0.37 | 0.31 | 0.27 | 0.25 |
| S.D. | 0.03 | 0.06 | 0.02 | 0.01 | 0.03 | 0.02 | 0.01 | 0.01 |

**Table S5. FRET-On Probabilities and Statistical Summary for Encapsulated Biosensors in Response to Varying Activated BoNT: Liposome Ratios.**

The table presents FRET-On probability values from three independent experiments, along with the calculated average (Avg.) and standard deviation (S.D.) for both proton and potassium sensors under acidic conditions at a decreasing activated two-chain BoNT/Ai to liposome ratio corresponding to Figure S5B.

| | Proton Sensor | | | | | Potassium Sensor | | |
|---|---|---|---|---|---|---|---|---|
| BoNT per Liposome | 50 | 25 | 10 | 5 | 50 | 25 | 10 | 5 |
| Exp. 1 | 0.94 | 0.74 | 0.34 | 0.24 | 0.79 | 0.62 | 0.33 | 0.32 |
| Exp. 2 | 0.91 | 0.55 | 0.30 | 0.27 | 0.77 | 0.66 | 0.42 | 0.34 |
| Exp. 3 | 0.65 | 0.53 | 0.43 | 0.22 | 0.84 | 0.70 | 0.48 | 0.32 |
| Avg. | 0.83 | 0.61 | 0.36 | 0.24 | 0.80 | 0.66 | 0.41 | 0.33 |
| S.D. | 0.13 | 0.10 | 0.05 | 0.02 | 0.03 | 0.03 | 0.06 | 0.01 |

**Table S6. FRET-On Probabilities and Statistical Summary for the Encapsulated Proton Sensor in Response to Varying Activated BoNT/Ai Concentrations.**

The table presents FRET-On probability values from three independent experiments, along with the calculated average (Avg.) and standard deviation (S.D.) for both proton and potassium sensors under acidic condition at a decreasing on-slide incubation concentration of the activated BoNT/Ai corresponding to Figure S6.

| BoNT Concentration (pM) | 400 | 200 | 100 | 50 | 25 | 10 | 0 |
|---|---|---|---|---|---|---|---|
| Exp. 1 | 0.51 | 0.40 | 0.27 | 0.16 | 0.07 | 0.03 | 0.02 |
| Exp. 2 | 0.44 | 0.37 | 0.27 | 0.19 | 0.10 | 0.05 | 0.02 |
| Exp. 3 | 0.44 | 0.38 | 0.31 | 0.16 | 0.09 | 0.03 | 0.01 |
| Avg. | 0.46 | 0.38 | 0.29 | 0.17 | 0.09 | 0.04 | 0.02 |
| S.D. | 0.03 | 0.01 | 0.02 | 0.02 | 0.01 | 0.01 | 4.6E-3 |

**Supporting References**

1. Band, P. A.; Blais, S.; Neubert, T. A.; Cardozo, T. J.; Ichtchenko, K. Recombinant Derivatives of Botulinum Neurotoxin a Engineered for Trafficking Studies and Neuronal Delivery. *Protein Expr Purif* **2010**, *71* (1), 62-73. DOI: 10.1016/j.pep.2009.12.013
